# Ternary semitransparent organic solar cells with a laminated top electrode

**DOI:** 10.1080/14686996.2016.1261602

**Published:** 2017-01-10

**Authors:** Mohammed Makha, Paolo Testa, Surendra Babu Anantharaman, Jakob Heier, Sandra Jenatsch, Nicolas Leclaire, Jean-Nicolas Tisserant, Anna C. Véron, Lei Wang, Frank Nüesch, Roland Hany

**Affiliations:** ^a^ Laboratory for Functional Polymers, Empa, Swiss Federal Institute for Materials Science and Technology, Dübendorf, Switzerland;; ^b^ ETH Zürich, Nanotechnology Group, Rüschlikon, Switzerland; ^c^ Institut des Matériaux, Ecole Polytechnique Fédéral de Lausanne, Lausanne, Switzerland

**Keywords:** Organic photovoltaics, ternary organic solar cells, transparent solar cell, lamination, PCBM, 50 Energy materials, 101 Self-assembly / Self-organized materials, 209 Solar cell / Photovoltaics

## Abstract

Tinted and colour-neutral semitransparent organic photovoltaic elements are of interest for building-integrated applications in windows, on glass roofs or on facades. We demonstrate a semitransparent organic photovoltaic cell with a dry-laminated top electrode that achieves a uniform average visible transmittance of 51% and a power conversion efficiency of 3%. The photo-active material is based on a majority blend composed of a visibly absorbing donor polymer and a fullerene acceptor, to which a selective near-infrared absorbing cyanine dye is added as a minority component. Our results show that organic ternary blends are attractive for the fabrication of semitransparent solar cells in general, because a guest component with a complementary absorption can compensate for the inevitably reduced current generation capability of a high-performing binary blend when applied as a thin, semitransparent film.

## Graphical abstract

**Figure:**
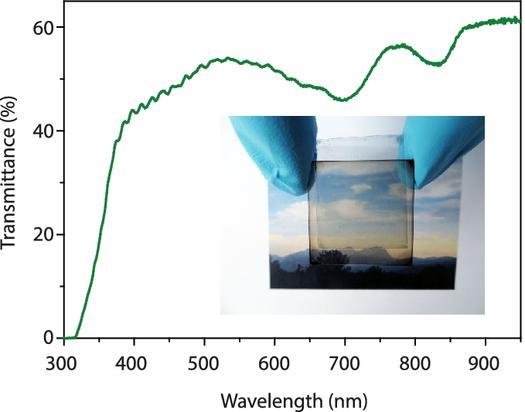

## Highlights

- A semitransparent ternary polymer:dye:fullerene organic solar cell is demonstrated.
- A flexible and transparent top electrode was applied via a dry lamination step.
- An average visible transmittance of 51% and a performance of 3% were achieved.

## 1. Introduction

Organic solar cells (OSCs) are interesting for providing low cost, large area and flexible energy conversion devices. Combining organic semiconductors with transparent and conductive electrodes allows for the fabrication of semitransparent OSCs. Such devices are building blocks for multijunction cells and are being intensively studied for applications in greenhouses, as chargers of portable electronics, or in building-integrated photovoltaics such as glass windows and roof covers [1–5].

Generally, the optimized photo-active layer thickness of OSCs is limited to below ~200 nm, and in many cases such thin organic films are inherently semitransparent. The level of transparency can be increased by decreasing the film thickness further; however, this is at the expense of the power conversion efficiency (PCE) because less light is absorbed by a thinner layer. Alternatively, organic semiconductors with discrete absorption bands can be used for the fabrication of coloured semitransparent or visibly transparent cells, if the absorption is outside of the visible wavelength range. A number of semitransparent OSCs with a high average visible transmittance (AVT) of over 50% and decent PCEs over 2% have been reported [6–9]. For lower acceptable AVT values, higher PCEs can be achieved [10]. However, we anticipate that in these cases the emerging perovskite solar cells might challenge OSCs, because several semitransparent perovskite cells with AVTs in the range of 20–40% and PCEs of ~6–11% have been shown only recently [11,12].

Here, we demonstrate semitransparent, inverted ternary OSCs with an AVT of 51% and a PCE of 3%. As majority components we used a binary blend of the donor polymer poly[(4,8-bis-(2-ethylhexyloxy)-benzo(1,2-b:4,5-b’)dithiophene)-2,6-diyl-alt-(4-(2-ethylhexanoyl)-thieno[3,4-b]thiophene-)-2,6-diyl)], PBDTTT-C, and the acceptor [6, 6]-phenyl-C_70_-butyric acid methyl ester, PC_70_BM [13–15]. Using this material system, opaque inverted solar cells with PCE of 6.7% have been reported [16]. Moreover, thin blended PBDTTT-C:PC_70_BM films exhibit a uniform absorption over the range of the visible spectrum, resulting in a greyish and colour-neutral appearance of semitransparent cells.

Decreasing the binary PBDTTT-C:PC_70_BM film thickness to enhance the AVT results in reduced current and associated PCE loss. To compensate for this loss, we added as a minority component the selective near-infrared (NIR) absorbing cyanine dye 2-[2-[2-chloro-3-[2-(1-ethyl-1,3-dihydro-3,3-dimethyl-2*H*-indol-2-ylidene)ethylidene]-1-cyclohexen-1-yl]ethenyl]-1-ethyl-3,3-dimethyl-3*H*-indolium (*OC*-6-11-∆)-tris[3,4,5,6-tetrachloro-1,2-benzenediolato(2-)-κ*O* ^1^, κ*O* ^2^]phosphate(1-), Cy7-T [17–20]. Cyanine dyes are characterized by a narrow, very intense absorption band. By increasing the number of double bonds the absorption maximum is shifted from the visible (mono-, tri- and pentamethine cyanines) to the NIR wavelength range (heptamethine cyanines). Heptamethine cyanines have been used for the fabrication of visibly transparent bilayer cyanine/C_60_ solar cells with AVT values over 60%; however, PCEs were limited to 2.2% [6,21,22]. For efficient harvesting of the complementary NIR light in the ternary system, the high extinction coefficient (> 2 × 10^5^ M^−1^ cm^−1^ at λ_max_ [6]) of the guest component Cy7-T is beneficial.

Ternary OSCs with multiple acceptor or donor materials have emerged as an effective strategy to overcome specific limitations of binary blend systems [14,23–27]. The general idea of ternary OSCs is to combine the advantages of the simple binary device fabrication with the extended absorption range of tandem cells. However, adjusting and understanding the electronic processes along with the developing microstructure of the film when adding a third minority component remains a big challenge. The presence of the guest can strongly impact the overall film morphology and thereby its location in the blend; this in turn affects the charge generation processes, that can take place via charge transfer, energy transfer, or a parallel linkage or alloy mechanism [25]. A ternary poly(3-hexylthiophene), P3HT:low band gap polymer:PCBM ([6, 6]-phenyl-C_61_-butyric acid methyl ester) blend was used in conjunction with silver nanowire electrodes for the fabrication of semitransparent OSCs [28].

To avoid the problem of optimizing the photoactive layer and transparent top electrode at the same time, we first adjusted the ternary material composition to achieve an acceptable compromise between the layer transparency and cell performance, and for this task we used an evaporated opaque MoO_3_/Ag back contact. In the second step, semitransparent cells were fabricated by using a transparent top electrode. For semitransparent solar cells, the transparent top conductor is a key factor that determines the device performance,[29,30]. and successful examples based on metal nanowire networks, thin metal films, carbon nanotubes or graphene have been demonstrated [6–8,10,28,31]. In our case, we used a flexible and transparent top electrode that was applied via a dry lamination step. The laminate electrode consists of a conductive plastic/metal mesh structure, coated with an adhesive mixture of poly(3,4-ethylenedioxytiophene)-poly(styrenesulfonate), PEDOT:PSS, and D-sorbitol. PEDOT:PSS/sorbitol films act as a conductive glue when heated above the melting point of sorbitol [32–35]. We optimized the process parameters to ensure intimate mechanical contact between the laminated cell parts and efficient hole transport to the current-collecting metal. The lamination process is simple and compatible with roll-to-roll systems for OSC production from solution.

## 2. Experimental details

Chemicals were purchased from commercial sources and were used as received. Cy7-T was synthesized according to the procedure in [6]. Pre-patterned indium tin oxide substrates (ITO, Geomatec (Yokohama, Japan), ~15 Ohms square^−1^) were subsequently cleaned in acetone, isopropyl alcohol, soap and deionized water. TiO_2_ films (50 nm) on ITO were prepared according to the procedure in [36]. Before deposition of the active layers, TiO_2_ coated substrates were heated for 10 min at 140 °C inside a glove box under N_2_ atmosphere (H_2_O < 1 ppm, O_2_ < 10 ppm). PBDTTT-C was purchased from Solarmer Materials (Beijing, China), and PC_70_BM from Solenne BV (Groningen, the Netherlands). Active binary blend layers on TiO_2_ were prepared by spin coating inside the glove box from dichlorobenzene (DCB) solutions containing PBDTTT-C (10 mg ml^−1^), PC_70_BM (15 mg ml^−1^) and 3 volume-% of diiodooctane (DIO). Film thicknesses were adjusted by varying the spin speed. For ternary blend layers, the PC_70_BM concentration was kept constant at 15 mg ml^−1^ DCB, and the total donor concentration was kept at 10 mg ml^−1^ with varying fractions of PBDTTT-C and Cy7-T. Also for these films, DIO was added. Ternary films were spin coated at 900 rpm for 60 s.

For opaque cells, 20 nm of MoO_3_ (99.9995%, Alfa Aesar (Karlsruhe, Germany)) and 80 nm of Ag (99.99%, Kurt J. Lesker, Jefferson Hills, PA, USA) were deposited by thermal evaporation at <5 × 10^−6^ mbar. Ag was evaporated through a shadow mask to define eight solar cells on one substrate with active areas of 3.1 and 7.1 mm^2^.

For optimized laminated cells, 5 nm of MoO_3_ were evaporated onto the active organic layer. Subsequently, a ~30 nm thick PEDOT:PSS Clevios HTL Solar (Heraeus, Leverkusen, Germany) layer was spin coated on MoO_3_ using a 5 μm filter outside the glove box and was then air dried for ~30 min. The laminate electrode consisted of a random mesh-like Ag network on PET substrate (Cima Nanotech, Oakdale, MN, USA, SANTE FS2000) that was planarized by spin coating a 1.3 μm thick layer composed of a mixture of PEDOT:PSS Clevios FCE (Heraeus) and 400 mg D-sorbitol (Sigma-Aldrich, St Louis, MO, USA, ≥ 98%) ml^−1^ PEDOT:PSS dispersion. The electrode was annealed at 120 °C for 10 min outside the glovebox and was laminated when still hot on the PEDOT:PSS HTL layer of the pre-fabricated cell using finger pressure. The bottom part was kept at room temperature during lamination with a typical area of 0.5 × 0.5 cm^2^.

For solar cell characterization, substrates were sealed in a vacuum tight box with current feedthroughs and an optical window that reflected 9% of the incident simulated AM1.5G solar radiation of 100 mW cm^−2^ from a calibrated solar simulator (Spectra-Nova, Ottawa, Canada). Cells were illuminated through the ITO/TiO_2_ cathode side only, and laminated cells were masked with a metal aperture of 0.2 cm diameter to define the active area. Reflection losses at the cell glass substrate were not considered in the calculation. At least six different cells were evaluated to obtain average PCE values in Tables 1 and 2. External quantum efficiencies (EQE) were measured on a commercial setup (Spequest, ReRa solutions BV (LOT-QuantumDesign, Romanel-sur-Morges, Switzerland)). The monochromatic light was chopped at 85 Hz and no bias light was applied during the measurement.

**Table 1.** Performance data of glass/ITO/TiO_2_/PBDTTT-C:Cy7-T:PC_70_BM/MoO_3_(20 nm)/Ag(80 nm) solar cells.

Entry	Ratio PBDTTT-C:Cy7-T:PC_70_BM (w/w/w)^a^	Active film thickness (± 5 nm)	V_oc_ (V)	J_sc_ (mA cm^−2^)	FF (%)	PCE ^b^(%)	PCE average (%)	AVT ^c^ 450–700 nm (%)
A	1:0:1.5	90	0.70	12	65	6.0	5.2 ± 0.6	56
B	1:0:1.5	60	0.68	7.4	54	3.0	2.9 ± 0.1	69
C	1:0:1.5	30	0.69	5.4	52	2.1	2.0 ± 0.1	75
D	0.7:0:1.5	83	0.69	8.7	71	4.7	4.5 ±0.2	66
E	0.7:0.3:1.5	95	0.71	10.4	68	5.5	4.7 ± 0.4	65
F	0:1:2 ^d^		0.71	4.1	38	1.1		

^a^ The concentration of PC_70_BM was constant at 15 mg ml^−1^.

^b^ The light intensity was 91 mW cm^−2^.

^c^ Average visible transmittance (AVT) values of the layer stack glass/ITO/TiO_2_/active layer/MoO_3_.

^d^ From [18].

**Table 2.** Performance data of semitransparent, laminated glass/ITO/TiO_2_/PBDTTT-C:Cy7-T:PC_70_BM (0.7:0.3:1.5 w/w/w)/MoO_3_ (X nm)/PEDOT:PSS HTL (Y nm)//PEDOT:PSS:sorbitol/Ag-mesh/PET solar cells.

Entry	Thickness MoO_3_ (nm)	Thickness PEDOT:PSS HTL (nm)	V_oc_ (V)	J_sc_ (mA cm^−2^)	FF (%)	PCE^a^ (%)	PCE average (%)
G	0	0	0.58	6.3	29	1.2	1.0 ± 0.2
H	5	0	0.62	8.4	46	2.6	2.1 ± 0.3
I	0	30	0.59	6.0	41	1.6	1.3 ± 0.2
J	5	30	0.59	8.8	53	3.0	
			0.56 ± 0.03^c^	8.1 ± 0.7	54 ± 4		2.7 ± 0.3
K	5 ^b^	30	0.59	6.6	50	2.1	

^a^ The light intensity was 91 mW cm^−2^.

^b^ Values when omitting Cy7-T.

^c^ Detailed average values from 20 cells.

Atomic force microscopy (AFM) measurements of ternary blends spin cast on glass were carried out in tapping mode on an MFP-3D (Asylum Research, Goleta, CA, USA) with Olympus AC160TS-R3 cantilevers. Indicated film thicknesses were measured using profilometry (Ambios XP1 from Ambios Technology, Santa Cruz, CA, USA) on reference samples that were coated on glass. Optical microscope images were taken with a 3D microscope (Leica DCM8 from Leica Microsystems, Heerbrugg, Switzerland). Transmission spectra were measured on a Varian Cary 50 UV-vis spectrophotometer (Agilent Technologies, Santa Clara, CA, USA) and air was defined as the baseline for all measurements. Contact angles were measured using a Krüss setup (Drop Shape Analyzer DSA 30, Krüss, Hamburg, Germany).

## 3. Results and discussion

**Figure 1.**
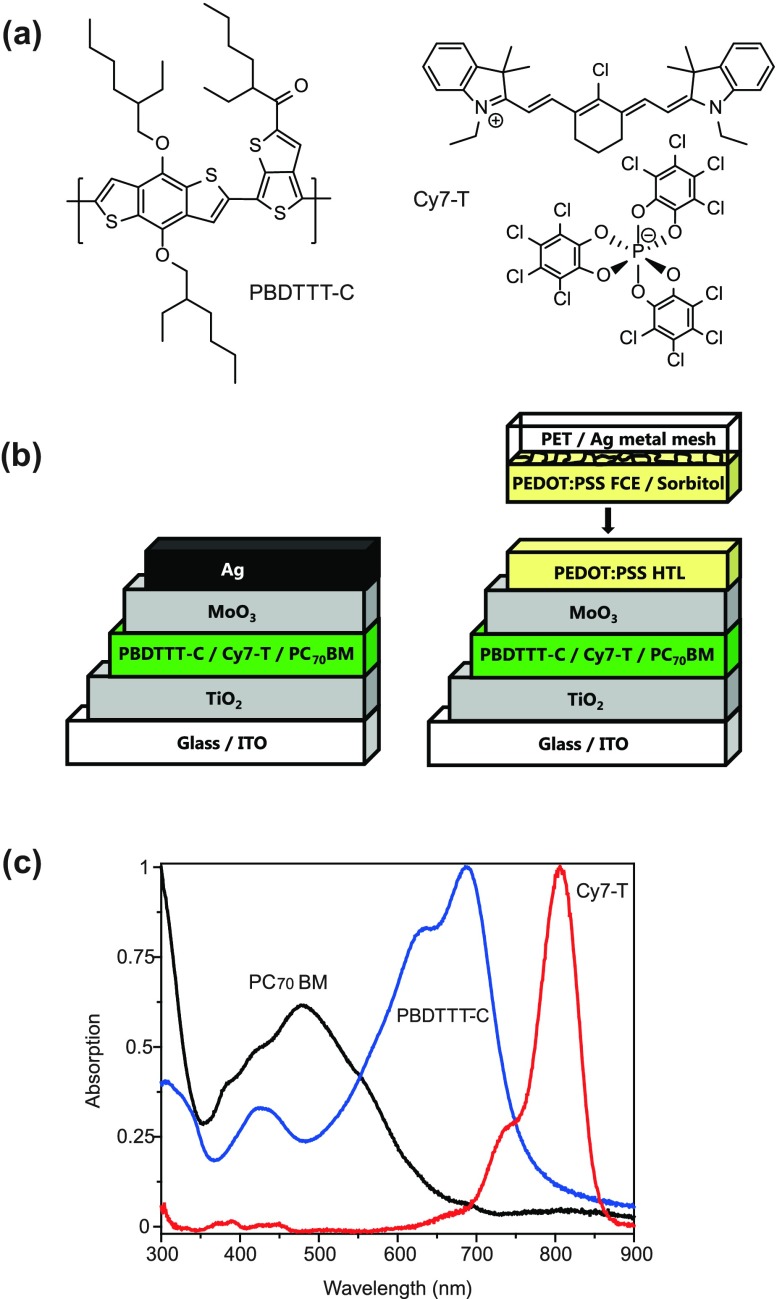
(a) Molecular structures of the polymer PBDTTT-C and the near-infrared absorbing cyanine dye Cy7-T with the anion ∆-TRISPHAT. (b) Schematic representation of the solar cell with an evaporated silver (Ag) top electrode (left) and a transparent laminated electrode (right). (c) Normalized absorption spectra (films on glass) of the electron donors and the acceptor material PC_70_BM.

The molecular structures of PBDTTT-C and Cy7-T, the opaque and semitransparent device architecture, and the film absorption spectra of the photo-active components are shown in Figure 1. Cy7-T has a complementary absorption to PBDTTT-C in the NIR region (λ_max_ = 820 nm on TiO_2_) and absorbs very little in the visible region.

**Figure 2.**
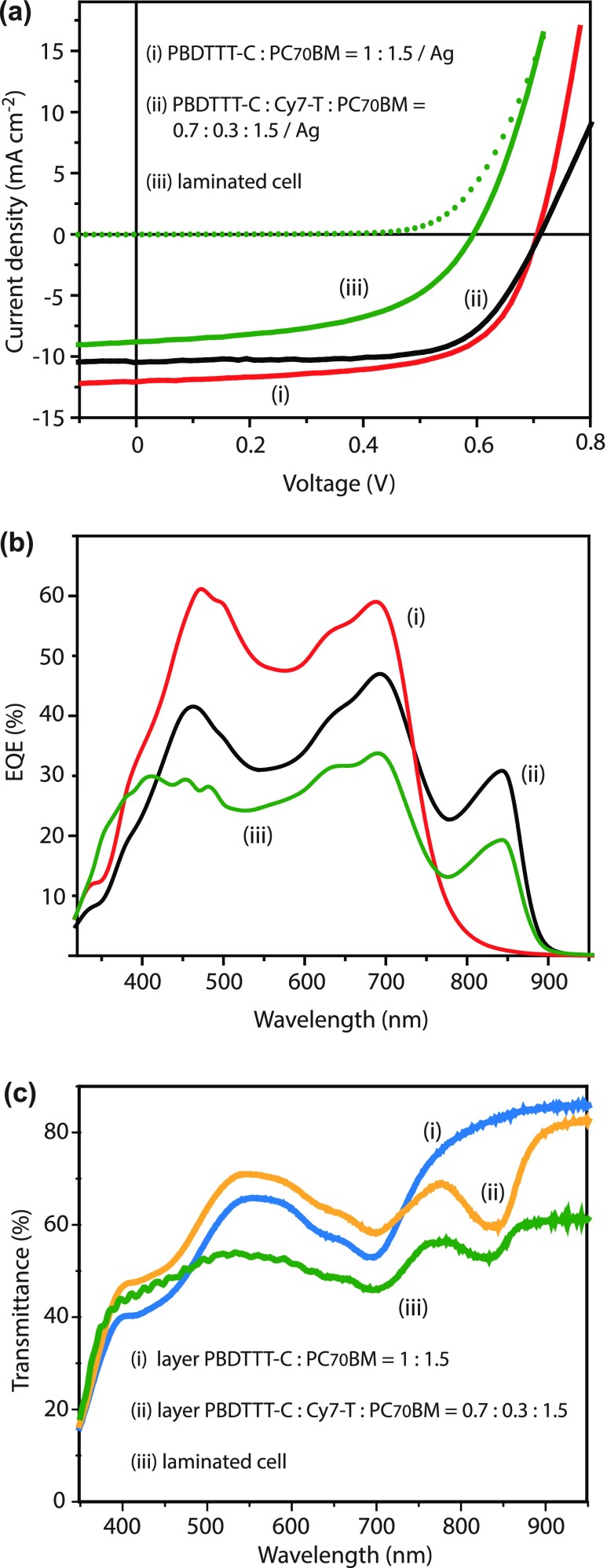
Best JV-scans (a) and typical external quantum efficiency (EQE) versus wavelength curves (b) for opaque (i) PBDTTT-C:PC_70_BM and (ii) PBDTTT-C:Cy7-T:PC_70_BM solar cells (Table 1, entries A and E, respectively). Short-circuit currents of (i) 11.8, (ii) 10.2 and (iii) 7.8 mA cm^−2^ were calculated by convoluting the EQE with the AM1.5G solar spectrum. (c) Transmittance spectra (i) and (ii) of the layer stacks glass/ITO/TiO_2_/active layer (~90 nm)/MoO_3_ with indicated weight fractions. In all parts (iii) indicates the optimized laminated cell (Table 2, entry J).

Using an opaque MoO_3_/Ag top electrode, optimized binary PBDTTT-C/PC_70_BM solar cells with a PCE of 6% could be fabricated (Table 1, entry A). The corresponding best JV scan and the EQE spectrum of a typical cell are shown in Figure 2. The film thickness was 90 nm and the AVT of the layer stack was 56%, measured next to the electrode (Figure 2(c)). Decreasing the film thickness stepwise to 30 nm increased the AVT up to 75%, accompanied with a drop of the short circuit current (J_sc_), as expected (Table 1, entry C). However, also the fill factor (FF) decreased continuously, resulting in a PCE of 2.1%. By considering that the J_sc_ decreases further when replacing the reflecting Ag with a semitransparent electrode, we concluded that it is not possible to fabricate semitransparent OSCs with PCE over 2% by simply reducing the thickness of the PBDTTT-C:PC_70_BM (1:1.5 w/w) film.

When resetting the photo-active layer thickness to ~90 nm, we found for a PBDTTT-C:PC_70_BM ratio of 0.7:1.5 w/w a good compromise between cell performance (PCE 4.7% with a high FF of 71%) and a high AVT of 66% (Table 1, entry D). To this mixture, Cy7-T was then added (Table 1, entry E). Because Cy7-T absorbs only slightly in the visible range, the AVT was not affected, but we measured a substantial J_sc_ increase (from 8.7 to 10.4 mA cm^−2^) and the PCE rose to 5.5% due to current generation in the NIR after light absorption by Cy7-T; the corresponding best JV and typical EQE curves are shown in Figure 2.

It is a general challenge to manipulate the performance of ternary OSCs in a predictable way. First, we note that the performance of optimized (1:1.5 w/w) binary PBDTTT-C:PC_70_BM cells could not be further improved by adding Cy7-T, in fractions of 0.1–0.5. The current peaked at a Cy7-T content of 0.15 with a ~15% relative J_sc_ increase, but the average PCE continuously decreased from 5.2% (no Cy7-T) to 3.7% (Cy7-T = 0.5) due to a drop in FF. We also screened ternary films with a high AVT, e.g. PBDTTT-C:Cy7-T:PC_70_BM = 0.5:0.5:1.5 and 0.3:0.7:1.5 w/w/w. These films showed AVT values of 67–71%, but again PCEs were limited to <3.3% due to a simultaneous reduction in J_sc_ and FF. In many reported systems small amounts of the third component cause a pronounced FF decrease [24].

**Figure 3.**
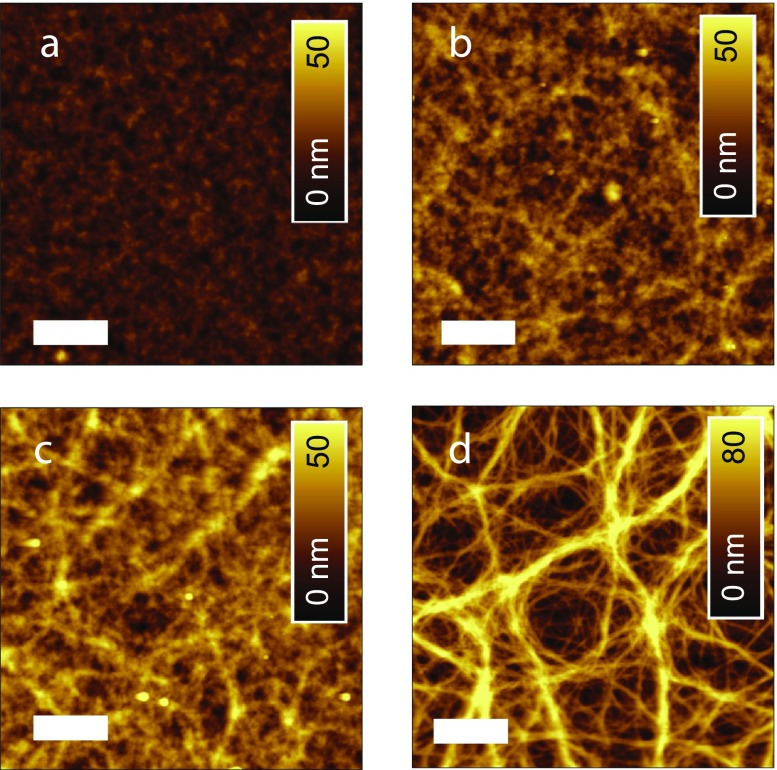
AFM topography of ternary PBDTTT-C:Cy7-T:PC_70_BM films with proportions (a) 1:0:1.5, (b) 0.7:0.3:1.5, (c) 0.5:0.5:1.5 and (d) 0.3:0.7:1.5. The scale bar is 1 μm.

Intimately connected with the electrical cell performance, we found that also the film morphology was sensitively dependent on the blend proportions (Figure 3) [26]. PBDTTT-C:PC_70_BM films showed a finely structured surface with a root mean square (rms) roughness of 3.5 nm. On a length scale of <500 nm, a network of fibrillar features is faintly observable [13]. When adding Cy7-T, the protruding fibrillary network grows (Figure 3(b–d)). The rms roughness of the optimized blend film (Figure 3(b)) was 6.2 nm. We assign the fibrils to the pure PBDTTT-C component, because the network was not dissolved when rinsing films with selective solvents (water, acetonitrile) for Cy7-T. Together with an earlier observation that Cy7-T and PCBM are partially miscible,[18] the AFM images allow to roughly sketch the phase separation behaviour of the ternary blend. The most important observation is that the polymer rich phase shows a totally different structure depending on the minority compound. The sample shown in Figure 3(a) is in a two-phase region expanding from the PC_70_BM/PBDTTT-C composition axis into the ternary phase space. Note: this sample does not contain any Cy7-T and is separating into a PC_70_BM rich and a PBDTTT-C rich phase [13]. The sample shown in Figure 3(d) is a ternary blend sample, which lies in the two phase region dominated by the Cy7-T and PBDTTT-C phase separation behaviour (expanding from the Cy7-T/PBDTTT-C composition axis into the ternary phase space). Differently from before, this sample phase separates into a pure PBDTTT-C phase (fibrillary morphology) and a blend phase of Cy7-T and PC_70_BM. Finally, samples shown in Figure 3(b) and (c) could be located either in the first two-phase region or in a three-phase region of the ternary diagram, not forming a pure polymer phase and thus obscuring the fibrillary structure. For solar cells intermixed blend phases are non-ideal; consequently, the Cy7-T:PC_70_BM binary system performed poorly (Table 1, Entry F).

**Figure 4.**
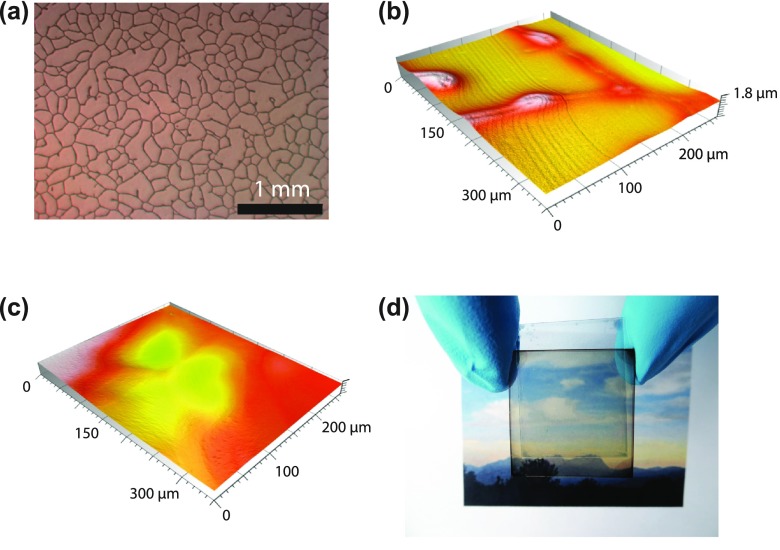
(a) Optical microscopy image of the commercial substrate that consists of a random mesh-like silver network on PET. Optical confocal microscopy images of the laminate electrode when coating with a ~450 nm thick (b) and ~1.3 μm thick (c) PEDOT:PSS:sorbitol film. For small film thicknesses (b), the metal network is not fully covered. (d) Photograph of the semitransparent, laminated cell.

Laminated, semitransparent OSCs were fabricated using 95 nm thick photo-active PBDTTT-C:Cy7-T:PC_70_BM (0.7:0.3:1.5 w/w/w) films. The laminate plastic substrate is covered with an irregular network of silver wires with linewidths of 4–12 μm and thicknesses of 0.7–1.3 μm (sheet resistance ~13 Ω square^−1^, Figure 4(a)). The full transmission of the substrate is ~94% for wavelengths above 450 nm. A PEDOT:PSS:sorbitol film (conductivity 1 S cm^−1^) of 1.3 μm was then coated onto the substrate, a thickness sufficient to cover the metal lines completely (Figure 4(b) and (c)). The laminate electrode had a full light transmission of ~85% above 450 nm. For cell assembly, the electrode was heated and dry laminated onto the pre-fabricated sub-cell that was kept at room temperature. The optimized recipe for sorbitol content, coated PEDOT:PSS:sorbitol film thickness and pre-annealing temperature of the laminate electrode was adopted from a recently developed process for the fabrication of perovskite solar cells [37].

Laminating the top electrode directly onto the photoactive layer resulted in a poor electrical contact with low FF (Table 2, entry G). Interfacial layers of 5 nm MoO_3_ (Table 2, entry H) or 30 nm PEDOT:PSS HTL (Table 2, entry I) increased the FF, and best results were obtained by using the combination of both MoO_3_ and PEDOT:PSS (Table 2, entry J). The presence of Cy7-T was also beneficial in semitransparent cells and J_sc_ dropped by more than 2 mA cm^−2^ when omitting the dye (Table 2, entry K). On the other hand, when replacing the laminate electrode by a reflecting Ag electrode, J_sc_ increased to 10 mA cm^−2^ and resulted in a PCE of 4.3% (V_oc_ = 0.67 V, FF = 59%).

Figure 4(d) shows a digital photograph of the semitransparent laminated cell (Table 2, entry J). The AVT value for this cell was 51% and the balanced transmittance in the visible (Figure 2(c)) results in a colour-neutral transparency perception. Since the sensitivity of the human eye is different for every visible wavelength, the human perception of transparency can differ from the radiometric AVT value. The visible light transmission (VLT) was calculated by integrating the transmission spectrum over the whole wavelength range, weighted by the product of photopic spectral response of the human eye and the AM1.5G solar spectrum [5]. A VLT value of 52% was obtained, in good agreement with the AVT value.

The specific beneficial role of the combined MoO_3_/PEDOT:PSS layer in our case is interesting because PEDOT:PSS // PEDOT:PSS laminated P3HT:PCBM OSCs without MoO_3_ have been demonstrated before [38,39]. When spin coating PEDOT:PSS HTL films on the photo-active layer we observed a rough surface with micrometre sized dewetting holes, while the wetting on MoO_3_ (rms roughness 1.8 nm) is much better, also confirmed by the contact angle of 11° for PEDOT:PSS HTL on a MoO_3_ film (data not shown). Therefore, MoO_3_ serves probably as a contact mediator between the ternary blend film and PEDOT:PSS HTL. This was further supported by a low cell FF when omitting MoO_3_ and using PEDOT:PSS HTL in combination with an evaporated Ag top contact (V_oc_ = 0.6 V, J_sc_ = 7.3 mA cm^−2^, FF = 38%).

In addition, we ruled out the possibility that MoO_3_ acts as protection layer during spin coating of PEDOT:PSS HTL and prevents the dissolution of Cy7-T. PBDTTT-C and PC_70_BM are insoluble in PEDOT:PSS, but when spin coating PEDOT:PSS HTL on a pure 20 nm thick Cy7-T film, around 20% of the dye is dissolved. Interestingly, no Cy7-T was dissolved from the ternary blend during coating with PEDOT:PSS HTL. We confirmed this observation with acetonitrile, an excellent solvent for pure Cy7-T. This can be indicative for the formation of an intermixed dye:PC_70_BM phase as postulated in the discussion of the phase behaviour (Figure 3) with a strongly altered solution behaviour.

## 4. Conclusions

Our results show that ternary systems are interesting candidates for the fabrication of semitransparent OSCs with AVT values over 50% and decent PCE (3%) because an NIR-absorbing guest material can effectively compensate the unavoidable current drop when the active film thickness of a high-performing binary blend is decreased. The challenge for ternary systems is to manipulate the electronic and morphological effects in a predictable way. Lamination of a semitransparent top electrode is a simple and roll-to-roll compatible method. The concept can be extended to other substrates with, for example, integrated barrier properties. For practical use, it is however appropriate to note that our thermally evaporated MoO_3_ interlayer should be replaced by a solution-based process in future work. This is because all other layers were coated from solution, and for cost-effective OSC fabrication the simultaneous use of vacuum and solution-based coating steps should be avoided [40].

## Disclosure statement

No potential conflict of interest was reported by the authors.

## Funding

This work was supported by Swiss Competence Center for Energy and Mobility [grant number Connect-PV]; Swiss National Science Foundation [grant number 152909, NRP70 PV2050 407040-153976/1, IZRJZ2_164179/1, 200021-144120/1, 157135].
